# m6A Modification in Non-Coding RNA: The Role in Cancer Drug Resistance

**DOI:** 10.3389/fonc.2021.746789

**Published:** 2021-10-20

**Authors:** Chen Chen, Yuying Guo, Yaxin Guo, Xiaoke Wu, Chaohua Si, Yanxin Xu, Qiaozhen Kang, Zhenqiang Sun

**Affiliations:** ^1^ Department of Colorectal Surgery, The First Affiliated Hospital of Zhengzhou University, Zhengzhou, China; ^2^ School of Life Science, Zhengzhou University, Zhengzhou, China; ^3^ Henan Institute of Medical and Pharmaceutical Sciences, Zhengzhou University, Zhengzhou, China; ^4^ School of Basic Medical Sciences, Zhengzhou University, Zhengzhou, China; ^5^ Department of Neurology, The First Affiliated Hospital of Zhengzhou University, Zhengzhou, China

**Keywords:** cancer drug resistance, m6A modification, miRNA, lncRNA, circRNA

## Abstract

Cancer drug resistance has always been a major difficulty in cancer therapy. In the face of drug pressure, resistant cancer cells show complex molecular mechanisms including epigenetic changes to maintain survival. Studies prove that cancer cells exhibit abnormal m6A modification after acquiring drug resistance. m6A modification in the target RNA including non-coding RNA can be a controller to determine the fate and metabolism of RNA by regulating their stability, subcellular localization, or translation. In particular, m6A-modified non-coding RNA plays multiple roles in multiple drug-resistant cancer cells, which can be a target for cancer drug resistance. Here, we provide an overview of the complex regulatory mechanisms of m6A-modified non-coding RNA in cancer drug resistance, and we discuss its potential value and challenges in clinical applications.

## Introduction

RNA modification determines cell fate by regulating gene expression and responds to environmental pressures (1, 2). m6A methylation is the most common modification in mammalian RNAs. Many studies showed that m6A sites are highly conservative and prefer to the motif RRACH (R = G, A, or U; R = G or A; H = A, C, or U) in human, yeast, and mice RNA (3–5). In the early days, m6A modification was reported to be present in ribosomal RNA and tRNA. Subsequent studies have found that there are also abundant m6A sites depositing in mRNA and non-coding RNA (6–8). Among them, m6A modification could affect these types of RNA metabolism events, including RNA cleavage, processing, transportation, stability, and translation by embedding dynamic and reversible deposition of m6A sites (9, 10). Then, the dysregulated mRNA or non-coding RNA was reported to play an important role in physiological and pathologic activities, such as cancer (11–13).

Cancer, which has still endangered human health, kills approximately 600,000 people each year (14, 15). Therefore, it is an urgent problem that scientists are committed to solving. Currently, five mainstream approaches for oncotherapy are surgical resection, radiotherapy and chemotherapy, targeted therapy, and biological immunotherapy. Among them, chemotherapy and targeted therapy are an important leap in the development of cancer therapy. However, it is unsatisfactory that therapy pressure compels the cancer cell appearing drug resistance (16). In brief, a group of cancer cells are inherently resistant or becomes resistant cancer cells under drug treatment (17). The molecular mechanism of tumor resistance is extremely complex and changeable (18). Many studies have shown that epigenetic changes, such as differential non-coding RNA and dynamic m6A modification, can lead to cancer drug resistance (2, 19).

In this review, we focused on the important role of m6A modification in cancer drug resistance, summarized the molecular mechanism of m6A-modified RNA including non-coding RNA involved in cancer drug resistance, and discussed the application and clinical value of m6A modification in the prediction and treatment of cancer resistance.

## Dynamic and Reversible Deposition in RNA by m6A Regulators

**Figure 1:**
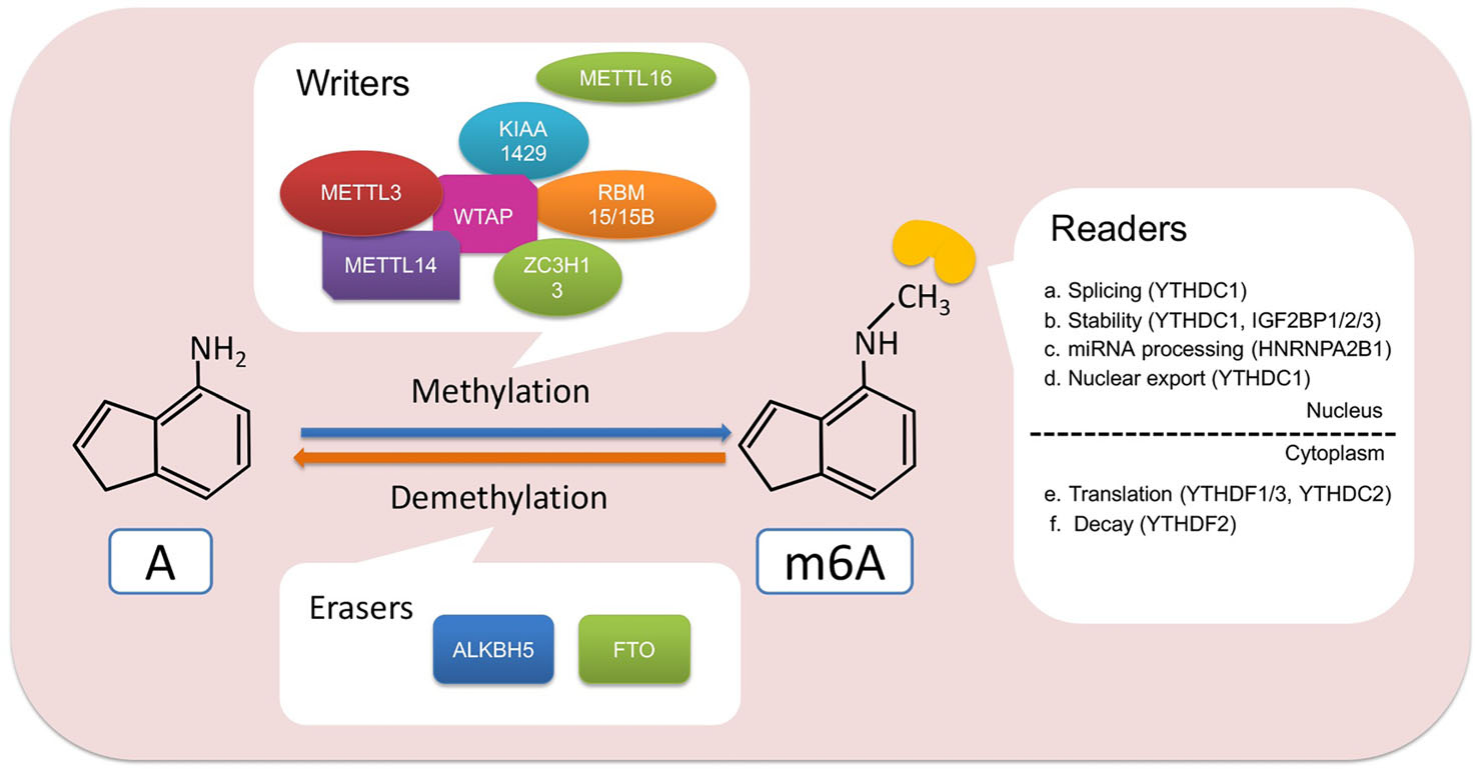
Regulators and functions involved in m6A modification. m6A writer, such as METTL3 and METTL14, binds to RNA to methylate adenine. Then, the m6A readers including YTHDC1/2, YTHDF1/2/3, IGF2BP1/2/3, and HNRNPA2B1 recognize the m6A site and participate in the process of RNA splicing, stability, nuclear transport, translation, degradation, or miRNA processing. In addition, m6A modifications can also be erased by FTO and ALKBH5, thereby affecting the fate of RNA. A, adenine.

Like other epigenetic modification, such as DNA methylation, m6A methylation is a dynamic and reversible phenomenon that embeds or removes m6A modification in RNA. This process is controlled by methylases and demethylases, which are vividly known as “writers” and “erasers” and then recognized by m6A “readers” (**Figure 1**). These m6A regulators affect multiple physiological and pathological activities such as cellular differentiation and cancer progression (10). The increasing rate suggested that m6A regulators decide RNA might play important roles in cancer progression (20).

### m6A Dynamic Deposition by m6A “Writers” and “Erasers”

Methyltransferase complexes, which consisted of multicomponents including methyltransferase-like 3/14/16 (METTL3/14/16), WT1-associated protein (WTAP), vir-like m6A methyltransferase-associated (VIRMA, another name: KIAA1429), zinc finger CCCH-type containing 13 (ZC3H13), and RNA-binding motif protein 15 (RBM15), is the key for catalyzing m6A sites in RNA. In the 1990s, an m6A complex containing three components was found in the mRNA of HeLa (21). Then, Joseph et al. found MT-A (MT-A70), which is also called METTL3, is the critical core of m6A methyltransferase complex and interacts with the methyl donor S-adenosylmethionine (SAM) for catalyzing methyl to partial RNA sequence (22–25). Moreover, another critical component of this complex, METTL14, could be as an RNA-binding platform stabilized METTL3 conformation when METTL3 is the catalytic core of this complex (23–25). WTAP, located at nuclear speckles, was another component that could interact with METTL3 and METTL14 for recruiting the m6A methyltransferase complex to RNA targets (26). Recently, Huang et al. reported that m6A deposition catalyzed by METTL3–METTL14–WTAP complex was regulated by histone H3 trimethylation at Lys36 (H3K36me3). Moreover, METTL14 could be a “reader” of H3K36me3 for promoting m6A deposition in transcribed nascent RNAs *via* binding adjacent RNA polymerase II (27). In addition, another m6A methylase component was identified in other types of RNAs, such as METTL16. Studies showed that m6A modification in U6 snRNA was catalyzed by METTL16 (28, 29). Moreover, METTL16 could efficiently induce splicing *via* binding 3′UTR of MAT2A, which encoded the SAM synthetase, a methyl donor (28). Removal of m6A modification in RNA was controlled by demethylase, FTO (obesity-associated protein), and alkB homolog 5 (ALKBH5). Initially, FTO was found to be correlated with childhood and adult obesity in early time (30). In 2011, FTO was firstly identified to have demethylation activity to decreased m6A amounts (31). Moreover, FTO could be as demethylase involved in physiology and pathology processes (32). ALKBH5 was identified as another demethylase in mammalian RNA and affects RNA export and RNA metabolism (33).

### The Regulation Mechanism of m6A Regulators *via* m6A “Reader”

Gene expression on the post-transcriptional level, including RNA splicing, transport, stability, and translation, was closely mediated by m6A modification *via* recruiting reader protein or changing RNA structure (34–38).

The newly transcribed RNA is regulated by the m6A regulator to affect its splicing. The METTL3–METTL14–WTAP complex was in nuclear speckles to regulate alternative gene splicing (26). In addition, m6A demethyltransferase FTO regulated splicing factor serine/arginine-rich splicing factor 2 (SRSF2)-targeted exons to control RNA splicing (39). Nuclear m6A “reader” YTH domain-containing protein 1 (YTHDC1) was reported to recruit SRSF3 and blocked SRSF10 to promote exon inclusion for affecting RNA splicing (40). Moreover, YTHDC1 mediated RNA export from the nucleus to the cytoplasm in HeLa cells *via* interacting with SRSF3, which is a splicing factor and nuclear export adaptor protein (41).

The fate of RNA in the cytoplasm is to be translated into protein or be degraded. Increasing studies proved that m6A reader YTH domain-containing family proteins (YTHDFs) greatly contributed to RNA translation (42). YTHDF1 promoted RNA translation efficiency *via* interacting with translation initiation factor complex 3 (eIF3) (38). Moreover, RNAs that modified m6A in its 5′UTR could be translated in a cap-independent manner by directly binding eIF3 (43). RNA stability protected by its 5′ cap and 3′ poly A tail is the key for RNA to execute its roles in the vital movement. When RNA was redundant, the RNA degradation mechanism was initiated by the deadenylation-dependent decay pathway and/or deadenylation-independent decay pathway. It has been reported that m6A modification destabilized m6A-embedded RNA (44, 45). YTHDF2 could recruit CCR4-NOT deadenylase complex for destabilizing m6A-containing RNA. This binding is critical for m6A-containing RNA deadenylated by CAF1 and CCR4 (37). Furthermore, YTHDF3 interacted with YTHDF1 to promote protein synthesis and regulated decay of methylated RNA in a YTHDF2-dependent manner (46). In addition, the insulin-like growth factor 2 mRNA-binding protein (IGF2BP) family, including IGF2BP1/2/3, could be an m6A “reader” to promote RNA stability by interacting with ELAV-like RNA-binding protein 1 (ELAVL1), matrin 3 (MATR3), and poly(A) binding protein cytoplasmic 1 (PABPC1) for regulating RNA translation under normal and stress conditions (47). HnRNP A2/B1, which contains two RNA recognition motifs, was another reported m6A “reader” for promoting microRNA (miRNA) processing (48). The above shows that m6A modification decided the RNA destiny regulated by “writers,” “erasers,” and “reader”.

## m6A Modification Contributes to Cancer Drug Resistance

**Figure 2:**
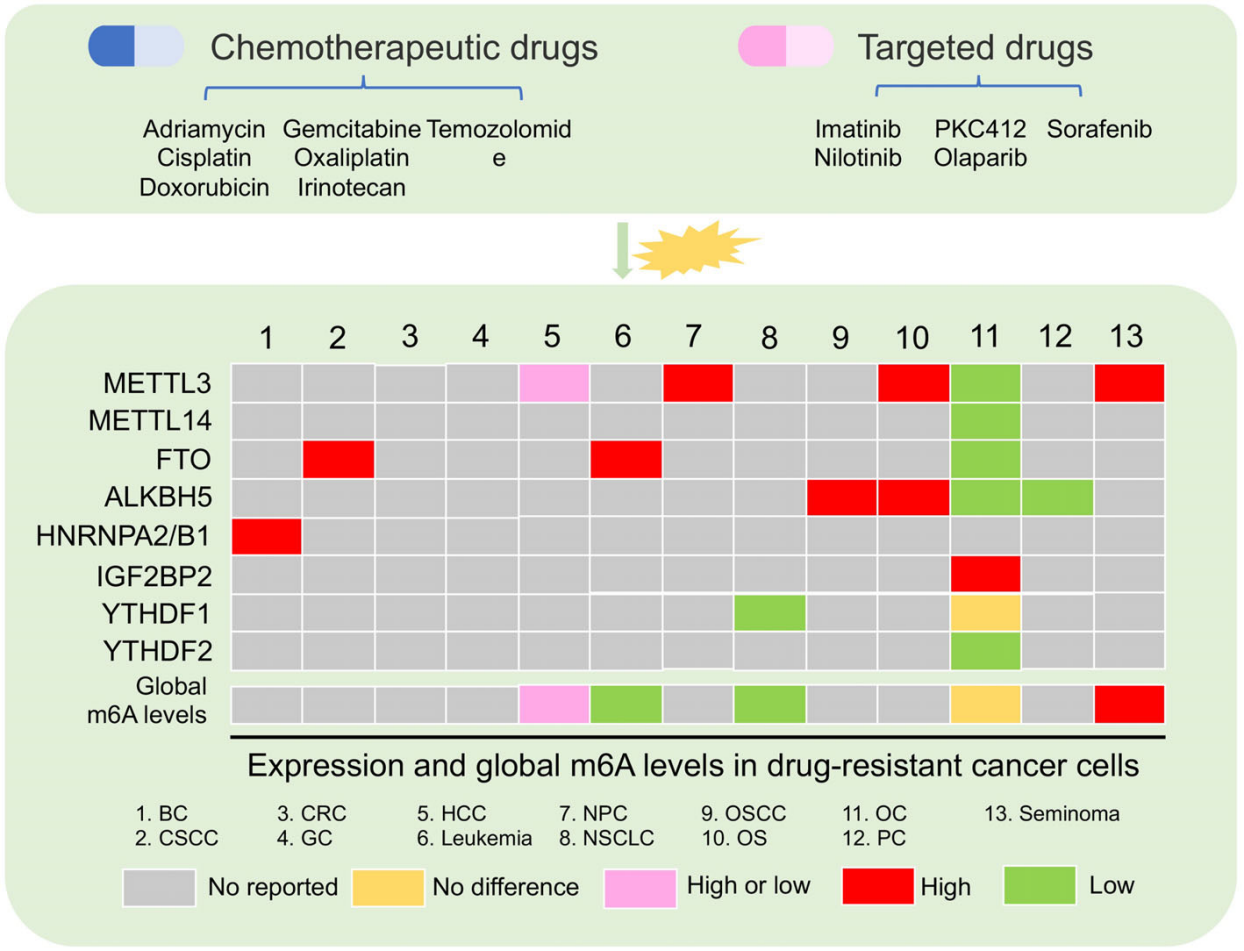
Aberrant m6A regulator and global m6A levels in drug-resistant cancer. The changes in m6A levels and the expression of m6A regulators were present after chemotherapy and targeted drug treatment in multiple cancer. BC, breast cancer; CSCC, cervical squamous cell carcinoma; CRC, colon cancer; GC, gastric cancer; HCC, hepatocellular carcinoma; NPC, nasopharyngeal carcinoma; NSCLC, non-small cell lung cancer; OSCC, oral squamous cell carcinoma; OS, osteosarcoma; OC, ovarian cancer; PC, pancreatic cancer.

Increasing data showed that m6A modification playing as a decision maker contributed to cancer progression (13). For example, overexpressed FTO is a critical oncogene in hematologic malignancies, such as AML (49, 50). High expression of METTL3 has been reported to be an oncogene in multiple solid tumors, such as hepatocellular carcinoma (HCC) (51), non-small cell lung cancer (NSCLC) (52), gastric cancer (GC) (53), colorectal cancer (CRC) (54), and bladder cancer (BLC) (55). Deregulation of m6A regulators in cancer cells is involved in cell stemness, proliferation, apoptosis, metastasis, immune, and drug resistance (13). Chemotherapy and targeted therapy are the main methods for patients with hematologic tumors and solid tumors. Cancer cells that develop resistance to drug treatment usually cause tumor recurrence, leading to a bad clinical outcome (16). Complicated mechanisms including epigenetic change are crucial for cancer cell obtaining resistance (56). Emerging data indicated that the global m6A level was abundant and aberrant in drug resistance of cancer cells (**Figure 2**) (57–59). Moreover, m6A “writers,” “erasers,” and “readers” were dysregulated in multiple cancer cells, and these regulators played important roles in cancer cells resisting chemotherapeutic drugs and targeted drug (**Table 1**) (57, 61, 63).

**Table 1:** The role and regulatory mechanism of m6A regulator in cancer drug resistance.

m6A regulator	Cancer type	Role in cancer	Expressions in cancer drug resistance	Drug	Target genes	Mechanism	Ref
ALKBH5	OSCC	Oncogene	High	Cisplatin	FOXM1	ALKBH5 promoted FOXM1 expression by demethylating its nascent transcripts	(60)
ALKBH5	OS	NA	High	Doxorubicin	NA	NA	(61)
ALKBH5	PC	Tumor suppressor	Low	Gemcitabine	WIF-1	ALKBH5 promoted WIF-1 transcription to hinder Wnt signaling	(62)
ALKBH5	OC	Oncogene	Low	Olaparib	FZD10	Silencing m6A demethylases ALKBH5, and FTO contributes to FZD10 upregulation	(57)
FTO	CSCC	Oncogene	High	Cisplatin	β-Catenin	FTO promoted gene expression of β-catenin *via* m6A modification	(63)
FTO	Leukemia	Oncogene	High	Imatinib, nilotinib, or PKC412	MERTK and BCL-2	m6A demethylated by FTO promoted MERTK and BCL-2 stability	(58)
FTO	OC	NA	Low	Olaparib	FZD10	Silencing m6A demethylases ALKBH5, and FTO contributes to FZD10 upregulation	(57)
HNRNPA2/B1	BC	NA	High	4-Hydroxytamoxifen, fulvestrant	MiR-29a-3p, miR-29b-3p, miR-222, miR-1266-5p, miR-1268a, and miR-671-3p	NA	(64)
IGF2BP2	OC	NA	High	Olaparib	NA	NA	(57)
METTL14	OC	NA	Low	Olaparib	NA	NA	(57)
METTL3	HCC	Oncogene	High	Adriamycin	ERRγ	Mettl3 delayed the half-life of precursor mRNA of ERRγ	(59)
METTL3	GC	Oncogene	NA	Cisplatin	ARHGAP5	ARHGAP5-AS1 recruits METTL3 to stimulate m6A modification of ARHGAP5 mRNA to stabilize ARHGAP5 mRNA	(65)
METTL3	NPC	Oncogene	High	Cisplatin	TRIM11	METTL3 promoted TRIM11 transcript stability *via* the m6A-IGF2BP2-dependent pathway	(66)
METTL3	NSCLC	Oncogene	NA	Cisplatin	YAP	METTL3 enhanced the translation of YAP mRNA by recruiting YTHDF1/3 and eIF3b	(52)
METTL3	Seminoma	NA	High	Cisplatin	TFAP2C	METTL3 enhances TFAP2C mRNA stability	(67)
METTL3	CRC	Oncogene	NA	Doxorubicin	p53	m6A modified by METTL3 promoted pre-mRNA splicing	(68)
METTL3	OC	Oncogene	Low	Olaparib	NA	NA	(57)
METTL3	CRC	Oncogene	NA	Oxaliplatin or irinotecan	CBX8	METTL3 enhanced CBX8 mRNA stability through a IGF2BP1-dependent mechanism	(69)
METTL3	HCC	Oncogene	Low	Sorafenib	FOXO3	METTL3 promoted FOXO3 stability through a YTHDF1-dependent mechanism	(70)
METTL3	OS	Oncogene	High	Doxorubicin	NA	NA	(61)
YTHDF1	OC	Oncogene	No difference	Cisplatin	TRIM29	YTHDF1 promoted TRIM29 translation	(71)
YTHDF1	NSCLC	Oncogene	Low	Cisplatin	Keap1	YTHDF1 promoted translational efficiency of Keap1	(72)
YTHDF2	OC	NA	Low	Olaparib	NA	NA	(57)

NA, not reported.

### Change of Global m6A Level Under Drug Pressure in Cancer

Increasing data showed that the global m6A level in cancer tissues was different from that in normal tissues (73, 74). Aberrant and abundant global m6A levels have also been reported in cancer drug resistance (57–59). In leukemia, researchers found that the development of resistance phenotype during tyrosine kinase inhibitor (TKI) treatment depended on the reduction of m6A detected by m6A dot-blotting due to FTO overexpression in leukemia cells. Furthermore, these results lead to the upregulation of proliferation/anti-apoptosis-related genes (58). Not only in blood tumors, but also in solid tumors, were there abnormal m6A levels in the drug-resistant cancer cell. For example, in HCC, low m6A global level cause by downregulated METTL3 was found in HepG2 cells resisting sorafenib therapy. This situation induced the increased autophagosomes of HepG2 cells to fight sorafenib therapy (70). Conversely, a high m6A global level in HepG2 cells and breast cancer cells with adriamycin resistance was detected by liquid chromatography coupled to tandem MS (LC-MS/MS) compared with parental cells. A high m6A level induced by upregulated METTL3 was responsible for the upregulation of ERRγ with metabolic reprogramming in chemoresistant cancer cells (59). In NSCLC, m6A but not the gene expression level in cisplatin-resistant A549 cells was significantly increased compared with A549 cells (72).

However, there are few studies that showed that the expression of m6A regulator was abnormal in the drug-resistant cancer cells, but the overall m6A level had no perturbation. In ovarian cancer, the global m6A level was not significantly changed in parental and cancer cells with olaparib and poly(ADP-ribose) polymerase inhibitor (PARPi) resistance. No change of global m6A levels in ovarian cancer resistant cells might be due to downregulated FTO, ALKBH5, METTL3, and METTL14. Furthermore, a high m6A level in 3′UTR region of the FZD10 mRNA due to the decrease of FTO and ALKBH5 was found to activate the Wnt signaling pathway, leading to PARPi resistance (57). These evidence indicated that the global m6A level is very complicated under different types of tumor cells and different therapy resistance. This complexity is due to the abnormal expression of different m6A regulators under resistant conditions. Therefore, it is necessary to acquaint entirely the molecular mechanism of abnormal m6A level and m6A regulators under the drug resistance pressure.

### Aberrant m6A Regulators in Cancer Drug Resistance

The global m6A level was regulated at different layers by “writers,” “erasers,” and “readers,” which showed irregular expression and played important roles in drug resistance of multiple cancers (**Table 1**).

### Aberrant m6A “Writers” in Cancer Drug Resistance

Dysregulated m6A “writers” were found in multiple cancers after drug treatment (57, 59, 61, 66, 70). It has been reported that METTL3 and METTL14 were downregulated in ovarian carcinoma cells (57). METTL3, as a critical methylase, was reported to play an essential oncogene in 2017 (75). It was not until recent years that increasing fact about the oncogene effect of METTL3 and its dysregulation in cancer drug resistance was displayed (76). In osteosarcoma (OS), METTL3 was found to have a critical role in OS cells through promoting cell proliferation, migration, and invasion ability (77). When OS cells showed doxorubicin resistance, an increasing METTL3 expression was detected (61). Similarly, highly expressed METTL3 in drug-resistant cell lines was also found in nasopharyngeal carcinoma (NPC) (61). A recent study showed that overexpression of METTL3 in NPC tissues promoted EMT process *via* m6A-modified Snail (78). Studies showed that high METTL3 expression in recurrent NPC tissue was associated with a bad prognosis of NPC patients (78, 79). After cisplatin induction, METTL3 was upregulated in drug-resistant NPC cell lines, where it could promote the viability of cell culture with a series of doses of cisplatin *via* regulating TRIM11 transcript (66). In HCC, METTL3 was found to promote HCC progression *via* YTHDF2-dependent silencing of SOCS2 on the post-transcriptional level (51). Interestingly, the role of METTL3 in HCC drug resistance was also complicated. Chen et al. reported that high METTL3 expression was detected in HepG2 cells with adriamycin resistance (59). Deleting METTL3 leads to the increase of dox sensitivity in adriamycin resistant HepG2 cells by regulating pre-mRNA of ERRγ. However, low METTL3 expression was found in human sorafenib-resistant HCC (70). Different from adriamycin, sorafenib is a multi-target drug that inhibited cancer cell proliferation and angiogenesis. This interesting result showed that METTL3 could be a tumor suppressor when its knockout could enhance sorafenib resistance, promote angiogenesis-associated genes expression, and activate pathway-associated autophagy in HCC cells under hypoxia condition (70). It is worth noting that METTL3 not only was involved in tumor progression but also plays an important role in tumor resistance, especially solid tumors.

### Aberrant m6A “Erasers” in Cancer Drug Resistance

As m6A demethylase, FTO and ALKBH5 were reported to play critical roles in multiple cancers and are involved in cancer drug resistance (58). FTO has been firstly reported m6A demethylase, which subsequently proved its oncogene role in AML (31, 50). Through reducing m6A abundance of ASB2 and RARA for destabilizing its transcripts, high FTO expression could enhance cell transformation and leukemogenesis, while it could inhibit all-trans-retinoic acid (ATRA)-induced AML cell differentiation (50). Moreover, high FTO expression was found in AMLs with multiple mutations including t(11q23)/MLL rearrangements, t (15, 17)/PML-RARA, FLT3-ITD, and/or NPM1. TKIs were considered for leukemia clinic treatment with these mutations (80, 81). However, rapidly acquiring resistance to TKIs was the main reason for the failure of leukemia treatment. Yan et al. found that FTO expression was increased in leukemia nilotinib-resistant cells. Upregulated FTO assisted leukemia cells to display more TKI resistant ability, and higher rates of cell growth *in vivo via* enhance mRNA stability of MERTK and BCL-2 (58). Moreover, FTO overexpression in cervical squamous cell carcinoma (CSCC) was proved to be related to chemo-radiotherapy resistance *in vitro* and *in vivo* by decreasing demethylation of β-catenin for promoting its expression (63). In addition, another m6A demethylase, ALKBH5, was upregulated in OS cell lines with doxorubicin resistance and doxorubicin resistance oral squamous cell carcinoma (OSCC) lines (60, 61). However, downregulated ALKBH5 expression was reported in a patient-derived xenograft (PDX) model treated with gemcitabine in pancreatic ductal adenocarcinoma (PDAC) (62). When ALKBH5 was overexpressed, PDAC cells were sensitive to chemotherapy due to a decrease of methylation in WIF-1 (62). Moreover, in ovarian cancer, downregulated FTO and ALKBH5 in PARPi resistance cancer cells also could be decreased PARPi sensitivity (57).

### Aberrant m6A “Readers” in Cancer Drug Resistance

The process that m6A modification mediated RNA metabolism always needs the participation of m6A “readers” to decide RNA fates. In cancer, aberrant reader proteins, such as YTHDF1, could lead to the disorders of RNA metabolism that contributed to tumor progression (82). The role of YTHDF1 was reported in various cancer types, and it plays a critical role in cancer (72, 83, 84). Cancer stem cells (CSCs) are cell populations with stem-like characteristics, which are considered to be the main cause of tumor chemoresistance and recurrence (85). A recent study reported that YTHDF1 promoted TRIM29 translation in cisplatin-resistant ovarian cancer cells. Decreasing YTHDF1 inhibited the CSC-like characteristics subsequently rescued by overexpressed TRIM29 (71). However, there is no difference of YTHDF1 in the cisplatin-resistant cells compared with the parental control cells, which need more clinical samples to be proved. Another study displayed that YTHDF1 was upregulated in NSCLC tissues compared with paracancerous tissues. Deletion YTHDF1 *in vitro* impeded cancer cell proliferation and inhibited cancer progression *in vivo* (72). Moreover, recent studies reported that YTHDF1 was involved not only in the glycolysis of cancer cells by promoting mRNA stability of PDK4 but also in cancer drug resistance (72, 86). A study showed that YTHDF1 expression was downregulated in cisplatin-resistant A549 cells. Silencing YTHDF1 rendered cancer cells resistant to cisplatin treatment, which showed a bad clinical outcome (72). In addition, decreased YTHDF2 expression and increased IGF2BP2 expression were found in resistant ovarian cancer cells, which might be contributing to FZD10 upregulation for promoting cancer drug resistance (57).

### Dysregulation of m6A-Modified RNA in Cancer Drug Resistance

**Figure 3:**
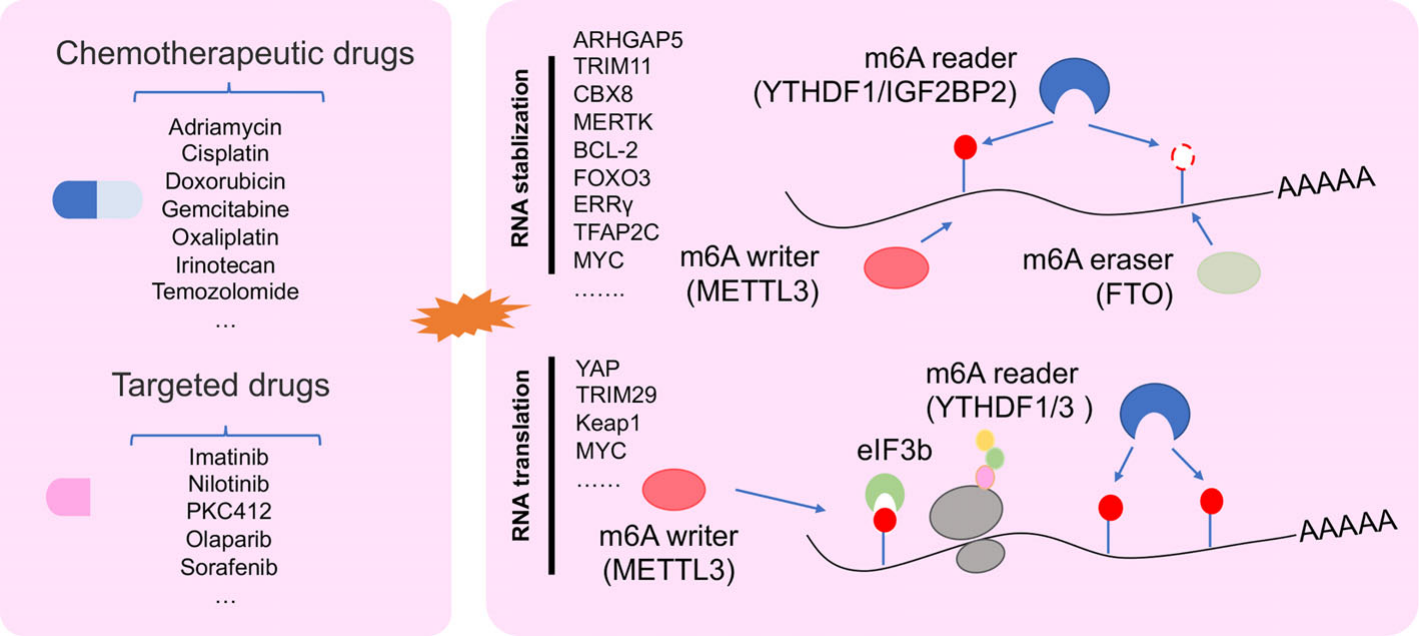
The regulatory mechanism of m6A regulator in drug resistant cancer. m6A modification in drug resistant cancer cell was involved in changing RNA stability, and RNA translation.

Increasing studies showed that dysregulation of m6A-deposited RNA significantly contributed to cancer progression (13). Recently, abundant m6A modification was found in 3′UTR, 5′UTR, and/or CDS in drug tolerance of cancer cells, such as ovarian cancer cell (57). m6A methyl embedded in different areas of RNA could influence RNA splicing, RNA stability, and translation in cancer drug resistance (**Figure 3**) (52, 58, 68).

### Changing RNA Stability for Cancer Drug Resistance

Transcript stability is a very important character for RNA to execute its function. Changes in RNA stability are the inevitable result of tumor progression (87). Increasing data showed that m6A participated in RNA stability for regulating cancer drug resistance mechanism. In drug-resistant NPC cells, METTL3 promoted TRIM11 transcript stability *via* the m6A-IGF2BP2-dependent pathway. m6A-marked TRIM11 could promote multidrug resistance and suppressed apoptosis by activating the β-catenin axis (66). Moreover, METLL3 enhanced RNA stability of CBX8, which could promote stemness and inhibit chemosensitivity by activating LGR5 transcription in colon cancer (CC) cells (69). Not only mRNA but also METTL3 could change the stability of pre-mRNA. A study reported that METTL3 could delay the half-life of pre-mRNA of ERRγ to enhance chemoresistance by upregulating ABCB1 and metabolic reprogramming in HCC (59). Demethylation of RNA regulated by ALKBH5 was found in cisplatin-resistant OSCC lines (60). A study showed that DDX3 could enhance CSC population by demethylation of FOXM1 and NANOG nascent transcript regulated by AKBH5 in chemoresistant cells (60). Other demethylase FTO could enhance RNA stability of MERTK and BCL-2 to control intrinsic and acquired resistance of CSCC *via* TKI therapy (58).

### Promoting RNA Translation for Cancer Drug Resistance

Dysregulation RNA translation was found in multiple diseases, including cancer (88, 89). Tumor cells trigger cellular stress under drug stress, such as genotoxic, oxidative, metabolic, and protein toxic stress. These stress responses lead to the plasticity of translational control, thereby changing tumor behavior that tend to drug resistance (90). m6A modification as a regulator could promote RNA translation *via* YTHDF1 interacting with initiation factor eIF3 under a selective regulation mechanism (38). Recent work reported that the deficiency of YTHDF1 inhibited translational efficiency of Keap1 and activated the antioxidant reactive oxygen species (ROS) clearance system (Nrf2-AKR1C1) under DDP therapeutic burden, which leads to a bad prognosis of NSCLC patients (72). Furthermore, METTL3 could be binding with YTHDF3, YTHDF1, and eIF3b to increase YAP1 translation for inducing DDP resistance and metastasis in NSCLC (52).

### Catalyze m6A Modification in Mutation Site for Cancer Drug Resistance

After drug therapy, cancer cells always accumulate DNA damage and increase the probability of gene mutations, which leads to a small number of tumor cells evolving drug resistance (91). Increasing or decreasing m6A level in RNA transcripts always leads to cancer progression. Recently, a study reported that m6A site mutation in transcripts may change m6A deposition and play an important role in cancer (92). TP53 mutation is a high-frequency mutation in 12 types of cancer and indicated close relation with clinical outcome (93). An interesting result showed that an m6A-modified mutation site found in the transited codon 273 of p53 pre-mRNA could increase the expression of R273H mutant protein and lead to multiple drug resistance of CRC (68). This showed that m6A site mutations in transcript greatly contributed to cancer drug resistance and could be a method to indicate patient prognosis in cancer therapy.

### Non-Coding RNAs Interacted With m6A Regulators in Cancer Drug Resistance

Non-coding RNAs are a class of RNAs that are not translated into protein and regulate the epigenetic change of genes. MiRNAs, long non-coding RNAs (lncRNAs), and circular RNAs (circRNAs) are three common non-coding RNAs that were reported to play important roles in cancer progression (94, 95). For example, highly expressed miR-21 was found to target PTEN for promoting HCC growth (96). LncTCF7 promoted self-renewal of HCC stem cells *via* triggering Wnt signaling (97). As the sponge of miR-9, circMTO1 promoted p21 expression for hindering HCC progression (98). Moreover, dysregulation of non-coding RNA in multiple cancers may be the primary cause of drug resistance (99–101). Linc00152, which promoted CRC progression, conferred resistance of oxaliplatin (L-OHP) that induced cancer cell apoptosis *via* the AKT pathway (102). In ovarian cancer cells, downregulated miR-29a/b/c enhanced the ability of cells to escape cell apoptosis induced by cisplatin *via* targeting collagen type I alpha 1 (COL1A1) (103). Furthermore, upregulated lncRNA UCA1 contributed to multiple drug resistance of GC *via* sponging miR-27b (104). Collectively, these findings displayed important roles of non-coding RNAs in one or more drug resistance of cancer. Interestingly, m6A modification commonly existed in not only mRNA but also non-coding RNA (9). More and more findings suggested a novel and complicated pattern of m6A regulator with non-coding RNA in cancer drug resistance.

### m6A Regulator–MicroRNA Model in Cancer Drug Resistance

MiRNAs are a group of highly abundant small RNAs (21–25 nucleotides) involved in post-transcriptional control *via* targeting 3′UTR of mRNA (105). It has been reported that miRNAs play a crucial role in cancer drug resistance (106). In NSCLC resistant to EGFR TKIs, exogenous miR-146b-5p in EGFR TKI-resistant cells could promote the cell apoptosis induced by EGFR TKIs *via* regulating the IRAK1/NF-κB pathway (107). In addition, miR-675-3p, which was from GC-secreted extracellular vesicles (GC-EVs), could enhance cisplatin resistance *in vivo via* targeting CXXC4 (108). These works displayed the potential role of miRNAs in cancer drug resistance.

**Figure 4:**
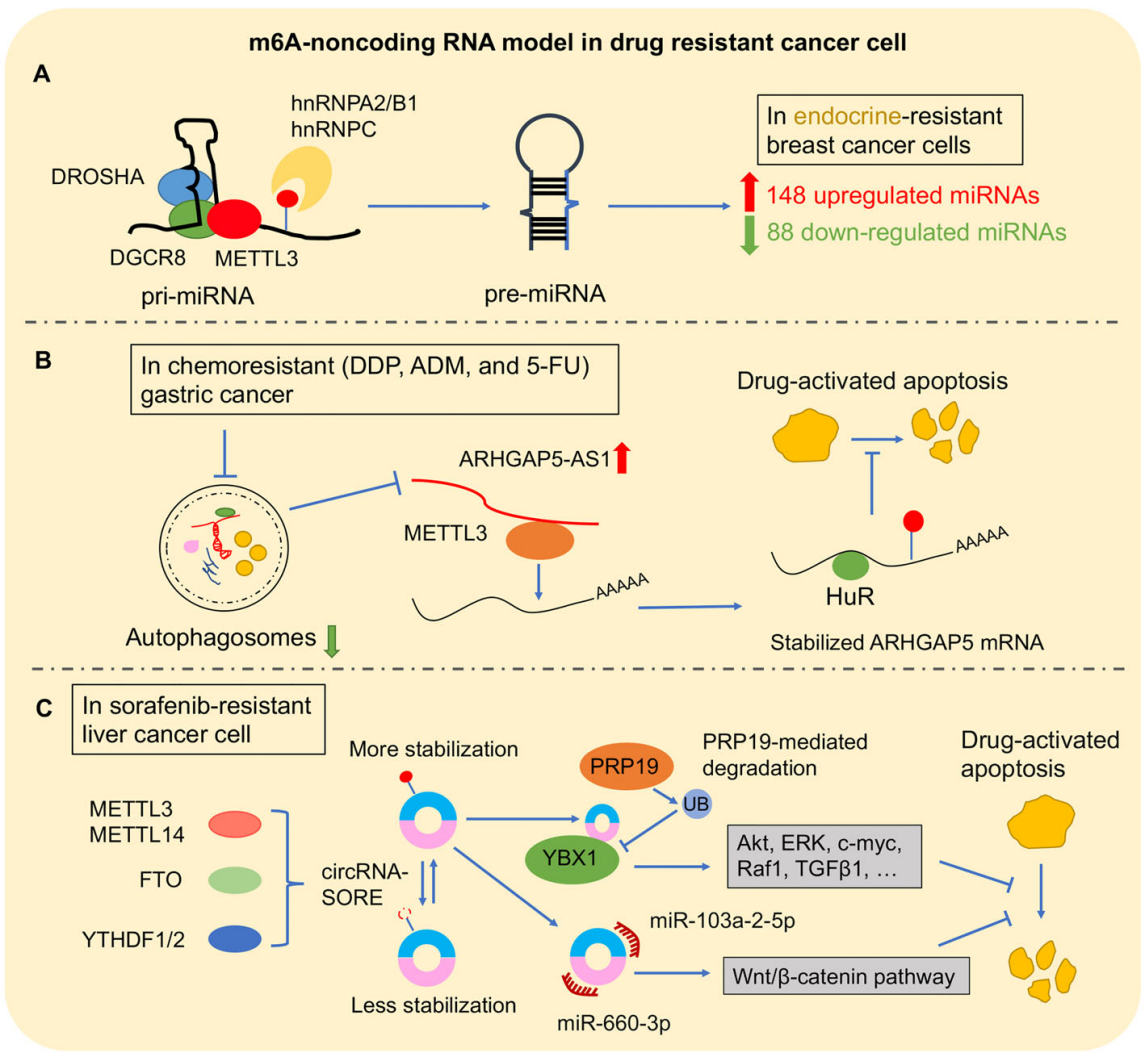
The m6A–non-coding RNA model in drug-resistant cancer. **(A)** m6A–miRNA model: m6A modification directly affects the pre-miRNA splicing, resulting in abnormal miRNA level changes in cancer drug resistance. **(B)** m6A–lncRNA model: ARHGAP5-AS1 recruits METTL3 to promote the stability of ARHGAP5 for inhibiting drug-induced apoptosis. **(C)** In sorafenib-resistant liver cancer cell, m6A modification promoted circRNA-SORE stabilization. Then, circRNA-SORE could directly bind YBX1 protein or sponge miR-660-3p and miR-103a-2-5p for arresting drug-activated apoptosis.

Recently, m6A modification was found in long primary miRNAs (pri-miRNAs) to affect miRNA processing. It is well known that pri-miRNAs are firstly transcribed in the nucleus. Subsequently, pri-miRNAs are transported into cytoplasm to be processed into precursor miRNAs (pre-miRNAs) *via* the double-stranded RNA-binding protein (RBP) DGCR8, which is the critical component of miRNA microprocessor complex binding with RNase III endonuclease DROSHA (109). METTL3 marked pri-miRNA for recognition and processing by DGCR8. Deleting METTL3 leads to the reduction of global miRNA and accumulation of unprocessed pri-miRNA. Gain of METTL3 reversed this change of global miRNA in a non-cell-type-specific manner, which suggested that m6A methylation plays a key post-transcriptional modification to promote miRNA mature (110). Moreover, m6A “reader” protein, heterogeneous nuclear ribonucleoprotein A2/B1 (hnRNPA2/B1), and hnRNPC could recognize m6A site in pri-miRNA and subsequently interact with DGCR8 to promote miRNA mature (48, 111). Interestingly, this new mechanism was found in the progression of multiple cancers (112–114). In BLC cells, METTL3 recruited DGCR8 to promote miR-221/222 maturation *via* decreasing PTEN expression for tumor growth (57). Another m6A complex component, METTL14, inhibited HCC metastasis *via* recognizing and binding DGCR8 to promote pri-miR-126 processing (115). Furthermore, m6A regulator–miRNA model was found to play an important role in cancer drug resistance (**Figure 4A**). In breast cancer cells, hnRNPA2/B1–miRNA model was reported to be involved in endocrine resistance. Upregulated hnRNPA2/B1 in endocrine-resistant breast cancer cells changed miRNA transcriptome including 148 upregulated miRNAs and 88 downregulated miRNAs. Moreover, overexpressed hnRNPA2/B1 decreased the sensitivity of cancer cells to 4-hydroxytamoxifen and fulvestrant (64). In addition, m6A regulators are involved in cancer drug resistance by changing miRNA expression by an indirect mechanism. MYC as a transcription factor is an oncogene by accelerating cell proliferation (116). A study reported that miRNA-155 (miR-155) and the miRNA-23a~27a~24-2 cluster (miR-23a cluster) were induced by MYC to promote tumorigenesis in glioma cells. Interestingly, FTO increased MYC stability and translation efficiency *via* wiping m6A modification, and its downregulation reduced the primary and mature transcripts of the transcripts of miR-155-5p, miR-24-3p, and miR-27a-3p (117). Furthermore, FTO inhibitor and MA2 increased the antitumor effect of temozolomide on decreasing the viability of glioma cells (117).

### m6A Regulator–Long Non-Coding RNA Model in Cancer Drug Resistance

Compared with miRNA, lncRNAs are a class of non-coding RNA with its length more than 200 nucleotides defecting coding protein function (118). LncRNA generally contributed to gene regulation *via* binding with miRNA, interacting with RBPs, or chromatin remodeling (119). In gemcitabine-resistant cells, GSTM3TV2 promoted the drug resistance by sponging let-7 for upregulating L-type amino acid transporter 2 (LAT2) and oxidized low-density lipoprotein receptor 1 (OLR1) in pancreatic cancer cells (120). Upregulated H19 in tamoxifen-resistant breast cancer cell line promoted autophagy by inhibiting the binding of DNMT3B to Beclin-1 (121). These studies showed that lncRNA could be a controller to regulate cancer drug resistance.

It has been observed to conserve m6A modification in lncRNA (122). Subsequently, increasing findings showed that m6A modification as “switches” regulated lncRNA–protein interaction contributed to the lncRNA–miRNA interaction or influenced lncRNA stability (52, 123, 124). Metastasis-associated lung adenocarcinoma transcript 1 (MALAT1) was a conserved lncRNA that was upregulated in multiple types of cancers (125). In NSCLC, MALAT1 promoted cancer cell migration *in vitro* and tumor formation and growth *in vivo*, and a high expression level of MALAT1 is associated with poor prognosis (126, 127). Recently, high m6A modification in transcripts of MALAT1 was observed (8). METTL3/YTHDF3 complex increased the stability of MALAT1, which sponged miR-1914-3p to promote the invasion and metastasis of NSCLC and enhanced sensitivity to DDP *via* regulating YAP1 (52). On the other hand, lncRNA was also involved in cancers when it was coupled with m6A regulators. Upregulated ALKBH5 in glioblastoma stem-like cells (GSC) demethylated FOXM1 to promote its expression. Interestingly, as a lncRNA antisense to FOXM1, FOXM1-AS accelerated the interaction between ALKBH5 with FOXM1 for promoting GSC proliferation and tumorigenesis (128). This interaction of lncRNA assistance to m6A regulators was also found in cancer drug resistance (**Figure 4B**). In chemoresistant GC cells, overexpressed ARHGAP5 antisense RNA 1 (ARHGAP5-AS1) promoted the cancer cell resistance to chemotherapeutic drugs including DDP, ADM, and 5-FU; decreased drug-activated apoptosis; and reduced intracellular drug concentration. ARHGAP5-AS1 could stabilize ARHGAP5 *via* recruiting METTL3 for m6A modification (65).

### m6A-Modified Circular RNA in Cancer Drug Resistance

CircRNAs are a class of non-coding RNA that has a special continuous loop by back splicing. The reported biological function of circRNA seems to bind miRNA or RBPs, regulate transcription, interfere with splicing, or translate peptide fragments and played important roles in cancer drug resistance (129, 130). In GC, circCUL2 could affect cisplatin sensitivity by inhibiting the autophagy activation mediated by miR-142-3p/ROCK2 (131). In glioblastoma cells that acquired temozolomide resistance, circASAP1 was markedly upregulated. Its overexpression promoted cell proliferation and temozolomide resistance by sponging miR-502-5p in glioblastoma (132). These studies showed that dysregulated circRNAs play a significant role in cancer drug resistance.

CircRNAs are widely modified by m6A and exhibited cell-type-specific methylation, which suggested the potentially important role of m6A modification in modulating circRNA biogenesis, transport, degradation, and translation (133, 134). It is well known that circRNAs are the product of back splicing, which is an alternative to linear splicing. Recently, m6A modification at specific sites was found to significantly modulate these two splicing ways. Furthermore, these specific m6A sites affected by METTL3–YTHDC1 were proved to command circRNA biogenesis, which offers a novel reason for circRNA biogenesis (135). CircRNA location is the key to determining the regulatory mechanisms of circRNA, especially ceRNA model in cytoplasm. However, few data illustrated the mechanisms of circRNA localization or nuclear export. It has been found that *Drosophila* DExH/D-box helicase Hel25E and human UAP49/56 act as key factors in the nuclear export of circRNAs depending on its length (136). Interestingly, m6A reader YTHDC1 was reported to accelerate cytoplasmic export of circNSUN2 in an m6A-dependent manner (137). The degradation of circRNA is another important part of understanding its function and expression, but the understanding of related mechanisms is still very limited. It has been reported that YTHDF2 interacting with HRSP12 acted as a guide of RNase P/MRP endoribonucleases for identifying and degrading m6A-modified circRNAs (138). In translation initiation, different from mRNA (cap-dependent pathway), circRNA lacking dissociative 5′ end was translated by cap-independent pathways, such as IRES-dependent pathway and m6A-dependent pathway (139–141). Yang et al. found that m6A-modified circRNAs have sufficient translation ability regulated by YTHDF3 and the translation initiation factors eIF4G2 and eIF3A (141). Recently, increasing findings prove the important role of the m6A-circRNA model in cancer progression. In CRC, m6A reader YTHDC1 expedited cytoplasmic export of circNSUN2 to promote cancer liver metastasis *via* stabilizing HMGA2, which acted as a driver of cancer metastasis *via* enhancing EMT process (137, 142, 143). In cervical cancer cells, an interesting study found that circE7 derived from human papilloma virus 16 (HPV16) translated into E7 protein to promote cancer cell proliferation in an m6A-dependent manner, which provided a basis for the diagnosis of high-risk HPV infection (144).

A recent study showed a significant role of m6A-circRNA model in HCC resistance with sorafenib, which is the first-line chemotherapeutic therapy for advanced HCC (**Figure 4C**). After sorafenib therapy, HCC cell with drug resistance showed high expression of circRNA-SORE in which depletion could enhance the cell-killing ability of sorafenib by stabilizing YBX1 (145). Another study showed that circRNA-SORE inhibited the efficacy of sorafenib-induced apoptosis in HCC cells. Interestingly, m6A modification resulted in the increased RNA stability of circRNA-SORE, which could sponge miR-103a-2-5p and miR-660-3p to activate the Wnt/β-catenin pathway (146).

## Implications for Cancer Drug Therapy

### Global m6A Level and m6A Regulators as a Diagnostic Approach

Tumor drug resistance is often the result of a combination of multiple mechanisms (147). Urgently, we need more effective assessment methods to judge whether patients have better treatment effects to improve patient prognosis. Researchers have found that methods such as gene sequencing for analysis of mutant genes, tumor-derived organoids for evaluating drug efficacy, or network-based machine learning can be used to predict tumor resistance (148, 149). Tiriac et al. reported that next-generation sequencing (NSG) technology combined with patient-derived organoid (PDO) drug classification can predict the response of pancreatic cancer patients and provide a basis for the selection of treatment options (150).

Abnormal and abundant global m6A levels frequently appeared in multiple cancer drug resistance. The global m6A level was decreased in leukemia cell line that obtained nilotinib and PKC412 resistance and HepG2 resisting sorafenib therapy, inversely upregulated in breast cancer cell with adriamycin resistance (58, 59). Therefore, the detection of m6A levels has the potential to be used as a method to predict whether tumor cells are/will be resistant. At present, the main technical methods used to detect the overall level of m6A are LC-MS/MS, colorimetry, and dot blotting (151). Based on liquid mass spectrometry, LC-MS/MS uses tandem MS to obtain molecular ion peaks and fragment ion peaks. LC-MS/MS can simultaneously perform qualitative and quantitative analyses of bases. With the use of a method similar to ELISA to suppress competitive immunity, the colorimetry uses m6A standards to quantify the m6A level of samples. Dot blotting uses m6A antibody to detect m6A level after binding RNA to a nylon membrane. Compared with the cumbersome operation of LC-MS/MS, colorimetry or dot blotting using a kit is simpler, faster, and more sensitive. These methods could have a potential application for detecting cancer drug resistance. Increasing data suggested that abnormally expressed m6A regulators can also be used as a detection strategy for drug response of multiple cancer. In NPC, m6A-modified TRIM11 stabilized by METTL3 could inhibit cancer cell apoptosis to promote multidrug resistance and enhance cisplatin resistance *in vivo* (66). Similar in GC, high METTL3 group tumors showed more sensitivity to mTOR inhibitor (everolimus) compared with the low METTL3 group (152). In ovarian cancer, downregulated FTO and ALKBH5 induced FZD10 upregulation, which led to reducing PARPi sensitivity (57). Moreover, in CSCC, FTO reduced m6A level of β-catenin to promote its expression for enhancing the chemo-radiotherapy resistance both *in vitro* and *in vivo* (63). Oppositely, FTO upregulation in leukemia showed more TKI tolerance *via* enhancing MERTK and BCL-2 stability (58).

### Targeting m6A Regulator–Non-Coding RNA Model

Increasing data displayed that m6A regulators play an important role in drug resistance of cancer, which suggested that m6A regulators may be a potential target to restore the drug sensitivity of tumor cells. In cisplatin-resistant seminoma cells, METTL3 promoted cellular viability by enhancing TFAP2C stability, which was identified by m6A reader, IGF2BP1. Encouragingly, more rapid tumor growth due to overexpressed METTL3 was inhibited by IGF2BP1 inhibition under cisplatin treatment *in vivo* (67). In NSCLC, METTL3 inhibition observably arrested tumor growth and enhanced sensitivity to cisplatin *in vivo* by reducing YAP1 expression (52). In addition, a significant antitumor drug effect was also found in drug-treated cancer cells combined with FTO inhibitors. In glioma, MA2, which could be an FTO inhibitor, notably inhibited cell proliferation compared with a single treatment group (117). Regarding the targeting of m6A-modified molecules, a variety of drugs have entered clinical research. For example, a competitive 2OG inhibitor can target and inhibit the activation of FTO. But it also inhibits other molecules, such as m1A demethylase ALKBH3, causing adverse side effects (153, 154). Many kinds of literature have confirmed that m6A modification participates in the regulation mechanism of tumor resistance. In detail, under the pressure of drugs, tumor cells cleverly change the modification level of m6A in RNA, thereby affecting the fate of RNA, especially non-coding RNA. Therefore, compared with looking for highly specific inhibitors of m6A regulatory molecules, directly targeting its downstream RNA molecules is also an ideal tumor drug resistance treatment policy. Non-coding RNA that interacts with m6A regulators has been reported to regulate the sensitivity of drug-resistant cells. A large number of studies have found that many non-coding RNAs can be used as targets or partners for m6A regulators in drug-resistant tumor cells. METTL3 coupled with lncRNA ARHGAP5-AS1 stabilized ARHGAP5, which promoted chemoresistance in GC (65). In addition, *in vivo* shRNA delivery of m6A-modified circRNA-SORE could enhance sorafenib efficacy in animal models (146). These studies suggested that m6A regulator–non-coding RNA model plays important roles in cancer drug resistance. In general, the combined strategy of using m6A targeted drugs with chemotherapy or its related non-coding RNA may provide new ideas for avoiding drug resistance in clinical practice.

### Potential Applications of m6A Regulation in Immunotherapy

Tumor immunotherapy is currently one of the mainstream methods of cancer treatment. However, the effects of immunotherapy in some tumors, such as lung cancer, are not satisfactory. The main reason is that the tumor behaved innately or acquired resistance to immunotherapy. Therefore, it is urgent to find a breakthrough in resistance to immunotherapy. Recently, most works implied m6A regulators have huge potential in PD-1 therapy. m6A regulators were reported to regulate PD-L1 expression, which implied its potential implication in immunotherapy. In intrahepatic cholangiocarcinoma (ICC), Qiu et al. found that tumor-intrinsic ALKBH5 stopped T-cell expansion and cytotoxicity by maintaining PD-L1 expression in tumor cells. Moreover, the results of clinical sample analysis from patients receiving anti-PD1 immunotherapy showed that strong nuclear expression patterns of ALKBH5 are more sensitive to anti-PD1 immunotherapy (155). Knockdown YTHDF1/2 in NSCLC cells could upregulate PD-L1 expression and multiple immune-related genes. High YTHDF1/2 expression showed a good prognostic outcome of NSCLC patients (156). Most notably, Han et al. found that the YTHDF1 could be a potential therapeutic target in immunotherapy because the absence of YTHDF1 can enhance the therapeutic effect of PD-L1 in Ythdf1−/− mice (157). Interestingly, non-coding RNA with m6A modification represented promising therapeutic targets in improving immunotherapeutic efficacy. m6A-modified circIGF2BP3 inhibited the activity of T cells *in vitro* and restrain antitumor immunity *in vivo* by upregulating PKP3 to elevate PD-L1 abundance. Furthermore, the inhibition of circIGF2BP3/PKP3 enhanced the effects of anti-PD-1 therapy in a Lewis lung carcinoma mouse model (158). These works suggested that m6A regulators or m6A-modified non-coding RNA could be a therapeutic target for immunotherapy in combination with PD-L1 inhibitors.

## Challenges and Future Perspectives

m6A modification is the most common modification in higher organisms. Many studies have confirmed that m6A modification plays important biological functions in mammals, such as regulating RNA stability, positioning, transportation, splicing, and translation at the transcriptional level. We have introduced some work that found that tumor cells exhibit abnormal m6A levels after drug resistance, and further control RNA stability, RNA translation, or regulation of non-coding RNA to avoid drug-induced apoptosis and escape successfully. These papers about cancer drug resistance displayed a part of the important role of m6A modification. Recent studies indicated that m6A modification was regulated by other factors. NADP, an enzyme-regulated metabolite, could directly bind FTO for improving its catalytic activity and adipogenesis (159). It indicated that some key regulators could be a potential strategy for regulating m6A modification, and we need more work to explore the mechanism that triggered abnormal global m6A level or differential expression of m6A regulators in the future.

At present, more and more m6A regulators are involved in clinical research for oncotherapy. However, their disadvantages cannot be ignored, such as poor specificity and side effects. Therefore, it is urgent to find another way to change the dilemma of m6A modification in the application of tumor resistance. Although m6A–non-coding RNA has positive prospects in clinical applications, the current situation shows that there are still shortcomings in the study of m6A–non-coding RNA mode. Firstly, m6A has fewer potential targets and partners in different drug resistance mechanisms of different tumors. Therefore, the regulatory mechanism of m6A modification in the common drug resistance of a variety of tumor cells still needs to be further explored more comprehensively. Secondly, there are fewer methods to detect the methylation level at the m6A site on the non-coding RNA sequence. Therefore, the method detecting more high resolution of m6A in non-coding RNA sequence needs to be exploited and widely apply in cancer drug treatment. Finally, the drug development of non-coding RNA related to m6A drug should be considered in the future.

## Conclusions

In the review, we summarized the research progress of m6A modification in cancer drug resistance. We show that the overall m6A level and m6A regulatory molecules in tumor cells are changed after different drug-resistant multiple tumors. For example, in adriamycin-resistant liver cancer cells, the expression of METL3 was significantly upregulated, resulting in a significant increase in overall m6A levels. However, in sorafenib-resistant liver cells, the expression of METL3 was significantly downregulated, resulting in a significant decrease in overall m6A levels. These contradictory results showed the complexity of m6A modification in drug-resistant cancer cells. In addition, we also summarized the complex regulatory mechanism of m6A modification in tumor resistance. Abnormal m6A regulators in tumor cells that have acquired drug resistance can maintain tumor cells against drug therapy by changing RNA stability, RNA translation, and catalytic mutation site m6A modification. Here, we focus on the important role of m6A modification that interacted with non-coding RNA in cancer drug resistance. Sorafenib is the first-line chemotherapeutic agent for the treatment of liver cancer. It is worth noting that circRNA-SORE stabilized by m6A modification can inhibit drug-activated apoptosis through a variety of ways, which elucidates that m6A regulator–non-coding RNA model plays an important role in cancer drug resistance. Therefore, targeting m6A-modified non-coding RNA has potential as a combined strategy to overcome therapeutic resistance. But the current situation is that the molecular understanding of m6A modification is still in its infancy. Therefore, more research is still needed to realize the application of m6A modification combined with non-coding RNA in cancer drug therapy.

## Author Contributions

Conceptualization, writing, and editing: all authors. All authors contributed to the article and approved the submitted version.

## Funding

This study was supported by the National Natural Science Foundation of China (81972663, U2004112); Key Scientific Research Projects of Institutions of Higher Education in Henan Province (19A310024); the National Natural Science Foundation of Henan Province (182300410342); and The Health Commission Technology Talents Overseas Training Project of Henan Province (2018140).

## Conflict of Interest

The authors declare that the research was conducted in the absence of any commercial or financial relationships that could be construed as a potential conflict of interest.

## Publisher’s Note

All claims expressed in this article are solely those of the authors and do not necessarily represent those of their affiliated organizations, or those of the publisher, the editors and the reviewers. Any product that may be evaluated in this article, or claim that may be made by its manufacturer, is not guaranteed or endorsed by the publisher.
